# Pyoderma Gangrenosum Presenting As Recurrent Vulvar Ulceration in a Nine-Year-Old Girl: A Case Report

**DOI:** 10.7759/cureus.112933

**Published:** 2026-07-18

**Authors:** Sofia De-La-O-Villalobos, Mariana Gonzalez-Plascencia, Ivan O Gomez-Figueroa, Adriana V Saenz-Ramirez, Raúl I Castillo-Cabrera, Ruben Rodriguez-Armendariz

**Affiliations:** 1 Internal Medicine, Hospital General “Presidente Lázaro Cárdenas del Río,” Instituto de Seguridad y Servicios Sociales de los Trabajadores del Estado (ISSSTE), Universidad Autónoma de Chihuahua, Chihuahua, MEX; 2 Hematology, Centro Medico Nacional 20 de Noviembre, Chihuahua, MEX; 3 Pediatric Medicine, Hospital General “Presidente Lázaro Cárdenas del Río,” Instituto de Seguridad y Servicios Sociales de los Trabajadores del Estado (ISSSTE), Universidad Autónoma de Chihuahua, Chihuahua, MEX

**Keywords:** neutrophilic dermatosis, pediatric dermatology, pediatric pyoderma gangrenosum, pyoderma gangrenosum (pg), vulvar ulceration

## Abstract

Pyoderma gangrenosum (PG) is a rare neutrophilic dermatosis characterized by rapidly progressive, painful ulcerations that often mimic infectious processes. Vulvar involvement is an uncommon presentation, particularly in pediatric patients, making the diagnosis especially challenging. We report the case of a nine-year-old girl who presented with rapidly evolving vulvar ulcerations that were unresponsive to intravenous and topical antibiotics. Because of the unusual clinical presentation and lack of response to antimicrobial treatment, a skin biopsy was performed, revealing dense neutrophilic dermatitis consistent with PG. An extensive evaluation failed to identify any associated systemic disease. After initiation of corticosteroid therapy, the patient showed marked clinical improvement, supporting the diagnosis of PG. This case describes an atypical pediatric presentation of vulvar PG and highlights the importance of considering PG in the differential diagnosis of persistent vulvar ulcerations. Early recognition may prevent unnecessary antimicrobial exposure, reduce diagnostic delays, and allow prompt initiation of appropriate immunosuppressive therapy.

## Introduction

Pyoderma gangrenosum (PG) is a rare neutrophilic dermatosis that presents with rapidly progressive, painful cutaneous ulcerations with undermined violaceous borders and surrounding erythema. Histopathologically, it is characterized by a dense sterile neutrophilic infiltrate in the absence of infection or primary vasculitis. Although the exact pathophysiological mechanisms are not yet fully understood, increasing evidence suggests that dysregulation of both innate and adaptive immune responses contributes to disease development [1,2].

A distinctive feature of PG is the pathergy phenomenon, in which minor trauma may trigger the formation of new lesions or exacerbate preexisting ones. This phenomenon has been described in approximately 20-30% of adult patients and represents an important diagnostic clue [1]. The clinical presentation is heterogeneous and ranges from isolated lesions to multiple concurrent ulcerations. According to clinical morphology, PG is classified into ulcerative, bullous, pustular, vegetative, and peristomal subtypes, with the ulcerative form being the most common [3].

PG most frequently affects the lower extremities, although lesions may also involve the trunk, upper extremities, head, and neck. The disease can occur at any age, but it predominantly affects adults in the fifth and sixth decades of life. Pediatric cases are uncommon, accounting for a small proportion of reported cases, and often demonstrate a wider and less predictable distribution than that seen in adults [4-6].

Because of its rarity and ability to mimic infectious, inflammatory, and malignant conditions, PG remains a diagnostic challenge, particularly in children and in unusual anatomical locations. We report the case of a nine-year-old girl with vulvar PG, an uncommon pediatric presentation that initially masqueraded as a refractory infectious process.

## Case presentation

A previously healthy nine-year-old girl presented with recurrent painful vulvar ulcerative lesions. She had no history of chronic medical conditions, allergies, previous surgeries, or regular medication use. She was exposed to passive tobacco smoke from both parents. Pubertal development was appropriate for her age, with thelarche and pubarche initiated and menarche not yet attained. Family history was notable for skin and liver cancer, type 2 diabetes mellitus, and renal disease in great-grandparents. Given the patient's age and the atypical location of the lesions, a multidisciplinary evaluation was performed to exclude sexually transmitted infections and the possibility of sexual abuse. Serologic testing for HIV, hepatitis B surface antigen (HBsAg), hepatitis C virus (HCV), *Treponema pallidum*, herpes simplex virus, and *Trypanosoma cruzi* was negative. In addition, the case was formally investigated by the appropriate child protection authorities, and no evidence of sexual abuse was identified.

**Figure 1:**
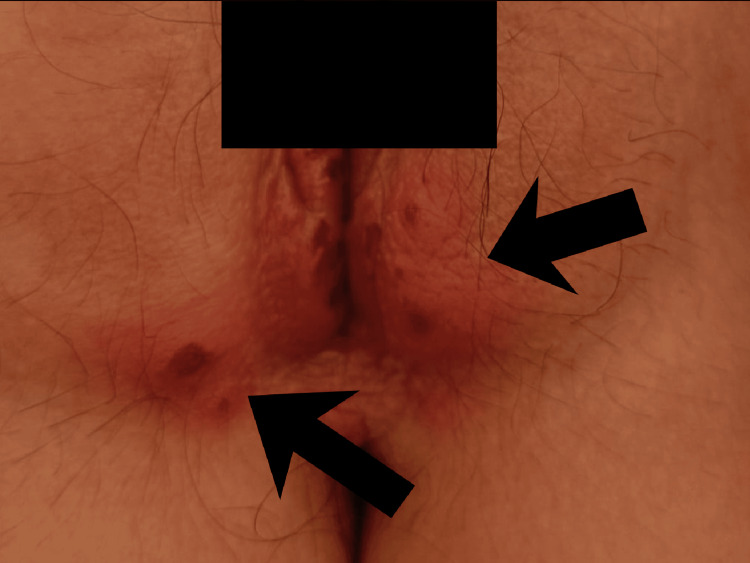
Initial clinical appearance of the vulvar lesions. Multiple asymmetric ulcerations of the labia majora with irregular borders and necrotic, exudative bases before diagnostic biopsy.

On physical examination, three to five painful vulvar ulcers were identified on the labia majora. The largest lesions measured approximately 3 mm, 3 mm, and 2 mm in diameter. The ulcers were asymmetric and irregularly shaped, with well-defined erythematous to violaceous borders and necrotic bases (Figure 1). The surrounding skin was tender to palpation. No purulent drainage, inguinal lymphadenopathy, or oral, perianal, or cutaneous lesions elsewhere on the body were identified.

According to the patient's parents, she had experienced a similar vulvar ulcer approximately one year earlier. At that time, no microbiological or virological studies were performed, and topical acyclovir was prescribed empirically because of suspected herpes simplex virus infection. The lesion resolved after treatment; however, approximately one year later, new ulcers developed at the same site with greater severity, characterized by increased pain, a higher number of lesions, and progressive clinical worsening. Associated symptoms included severe localized vulvar pain exacerbated by friction from clothing, intermittent spontaneous self-limited bleeding, and febrile episodes reaching 39°C.

Laboratory investigations are summarized in Table 1. Studies revealed marked leukocytosis with neutrophilia. Thyroid function tests demonstrated subclinical hypothyroidism, and total IgE levels were elevated. An extensive infectious workup, including serologic testing for HIV, HBsAg, HCV, *Treponema pallidum*, herpes simplex virus, and *Trypanosoma cruzi*, was negative. Initial urinalysis findings were suggestive of a possible UTI; therefore, empirical treatment with intravenous ceftriaxone was initiated while awaiting urine culture results. The urine culture subsequently showed no bacterial growth. However, the vulvar ulcers persisted without clinical improvement, supporting the exclusion of an infectious etiology and favoring a noninfectious inflammatory process.

**Table 1:** Laboratory and microbiological findings at presentation. Findings included leukocytosis with marked neutrophilia, urinary abnormalities, mildly elevated thyroid-stimulating hormone with normal total T3 and T4 levels, elevated immunoglobulin E (IgE), and negative serologic testing for HIV, HBsAg, HCV, *Treponema pallidum*, HSV, and *Trypanosoma cruzi*. ANC: Absolute neutrophil count; TSH: Thyroid-stimulating hormone; T3: Triiodothyronine; T4: Thyroxine; IgE: Immunoglobulin E; HPF: High-power field; HBsAg: Hepatitis B surface antigen; HCV: Hepatitis C virus; HSV: Herpes simplex virus.

Category	Parameter	Result	Units	Pediatric reference range (9 years)
Complete blood count	WBC	29,130	cells/µL	4,500-13,500
	ANC	24,810	cells/µL	1,500-8,000
Urinalysis	Protein	25	mg/dL	Negative to trace
	Erythrocytes	250	cells/µL	<5 RBC/HPF
	Leukocytes	40-50	cells/HPF	0-5 WBC/HPF
	Bacteriuria	Moderate	-	Absent
Thyroid function	TSH	5.39	µIU/mL	0.6-4.8*
	Total thyroxine (T4)	9.76	µg/dL	5.5-12.8
	Total triiodothyronine (T3)	1.22	ng/mL	0.9-2.3
Immunologic studies	Total immunoglobulin E (IgE)	513	IU/mL	<90-100†
Infectious workup	HIV serology	Negative	-	Negative
	Hepatitis B surface antigen (HBsAg)	Negative	-	Negative
	HCV antibody	Negative	-	Negative
	*Treponema pallidum* serology	Negative	-	Negative
	Herpes simplex virus (HSV-1/2) serology	Negative	-	Negative
	*Trypanosoma cruzi* serology	Negative	-	Negative

**Figure 2:**
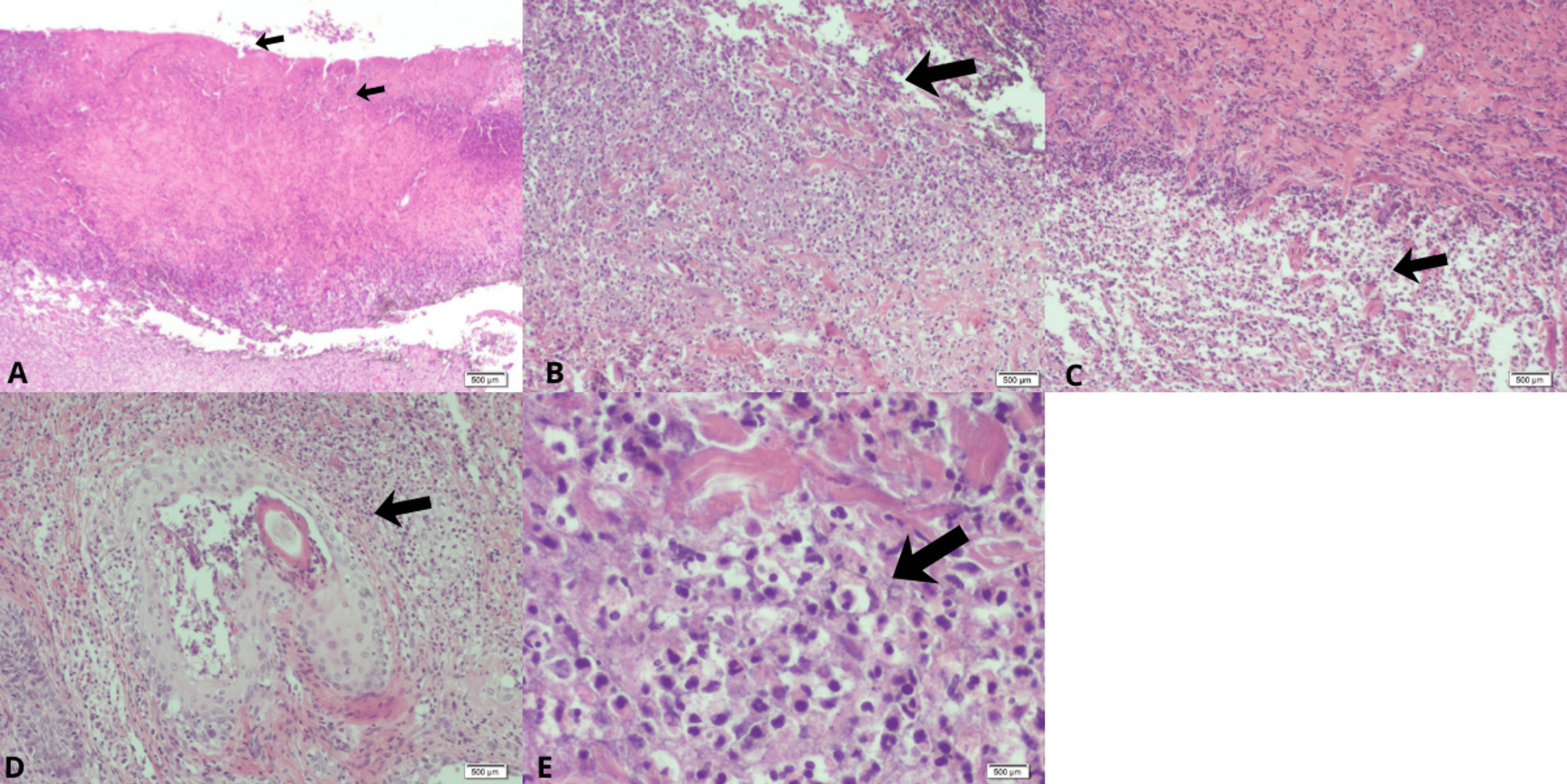
Histopathologic findings of vulvar pyoderma gangrenosum. H&E staining. (A) Parakeratosis, spongiosis, and neutrophilic exocytosis (×4). (B) Extensive epidermal ulceration (×10). (C) Dense neutrophilic infiltrate extending from the dermis into the subcutaneous tissue (×10). (D) Mixed inflammatory infiltrate composed of lymphocytes and histiocytes (×20). (E) Follicular destruction with prominent neutrophilic infiltration (×40).

Due to diagnostic uncertainty, a spindle-shaped incisional biopsy measuring 0.8 × 0.6 × 0.4 cm was obtained from the vulvar ulcerative lesion. Histopathologic examination revealed a small fragment of epidermis with parakeratosis, spongiosis, and neutrophilic exocytosis (Figure 2A). Most of the epidermis was ulcerated (Figure 2B), with a dense neutrophilic exudate containing pyknotic debris and fibrin extending into the superficial and reticular dermis and reaching the hypodermis (Figure 2C). The reticular dermis also demonstrated a mixed inflammatory infiltrate composed of lymphocytes and histiocytes (Figure 2D). In the superficial dermis, hair follicles were destroyed by the inflammatory exudate, with residual follicular structures infiltrated by neutrophils and pyknotic cells (Figure 2E).

These findings were consistent with ulcerative neutrophilic dermatosis, suggestive of PG in clinicopathologic correlation. No evidence of vasculitis or a specific infectious etiology was identified. The diagnosis of PG was supported by the characteristic clinical presentation, exclusion of infectious etiologies, histopathologic findings of neutrophilic dermatosis, and favorable response to corticosteroid therapy. Treatment with oral prednisone at 1 mg/kg/day combined with topical pimecrolimus resulted in an approximately 50% reduction in the lesions within three weeks.

## Discussion

PG is an uncommon neutrophilic dermatosis in children, and vulvar involvement is rare, making its recognition particularly challenging in the pediatric population. This case highlights the need to include PG in the differential diagnosis of rapidly progressive vulvar ulcerations in children, particularly when lesions do not improve despite appropriate antimicrobial treatment.

PG often begins as a painful inflammatory papule, pustule, or nodule on an erythematous and edematous base. These early lesions progress rapidly, undergoing tissue necrosis and ulceration. Established ulcers are characterized by irregular, undermined borders with a violaceous appearance and a surrounding zone of inflammation. The ulcer bed frequently contains purulent exudate or fibrin during the acute phase and may later develop abundant granulation tissue [2].

The most affected sites in pediatric patients are the trunk and lower extremities, each involved in approximately 77% of patients, followed by the upper extremities and the head and neck region, each reported in 38% of cases [7]. Adult PG typically exhibits a predilection for the anterior lower legs, attributed to the higher frequency of minor trauma in these areas via the pathergy phenomenon. Previously reported pediatric cases of PG have shown heterogeneous anatomical involvement, including the extremities, trunk, face, oral mucosa, and genital region (Table 2).

**Table 2:** Previously reported pediatric cases of pyoderma gangrenosum and anatomical sites of involvement. PG: Pyoderma gangrenosum. References correspond to the numbering used in the manuscript.

Reference	Age	Sex	Anatomical site of involvement	Clinical characteristics
Medeiros CC et al. [8]	9 months	Female	Right lower extremity (leg)	Ulcerative lesions of the leg; infectious studies negative; complete healing after systemic corticosteroids
Lambropoulos V et al. [9]	13 years	Female	Left ankle (post-traumatic), left arm (venipuncture site)	Pediatric PG associated with pathergy after orthopedic surgery and vascular access
Browning J et al. [10]	10 years	Female	Vulvar region	Pediatric vulvar PG with painful ulcerations and systemic inflammatory findings
Sarma N et al. [11]	12 years	Female	Multiple sites, including the scalp, face, ear, trunk, buttocks, thighs, legs, hands, and feet	Extensive ulcerative PG without mucosal involvement; no underlying systemic disease identified
East-Innis A et al. [12]	2 years	Female	Lower extremity	PG associated with osteomyelitis
Crouse L et al. [13]	11 months	Female	Face, trunk, buttocks, bilateral thighs, upper and lower extremities, hands, and feet; oral mucosal involvement	Infantile PG presenting with papules, pustules, erosive plaques, and progressive ulcerations
Schoch JJ et al. [7]	<18 years (n=13)	Predominantly female (62%)	Trunk (77%), lower extremities (77%), upper extremities (38%), head and neck (38%)	Pediatric cohort describing common anatomical distributions and associated diseases
Botello Mojica HM et al. [14]	6 years	Male	Trunk, lower extremities	Generalized pediatric PG with multiple ulcerative lesions
Present case	9 years	Female	Vulva (labia majora)	Recurrent vulvar ulcers with fever, neutrophilia, negative infectious workup, and biopsy findings compatible with PG

PG encompasses several clinical subtypes, each with distinct morphological characteristics. The most common presentation in both adults and children is the ulcerative form, which typically presents as painful necrotic ulcers with violaceous, undermined borders. Other recognized variants include bullous, vegetative, peristomal, and postoperative PG, which differ in their clinical appearance, anatomical distribution, and degree of association with systemic disorders [3].

Histopathological findings in PG are nonspecific but may provide supportive evidence for the diagnosis. Typical features include dermal edema and a dense neutrophilic infiltrate with suppurative inflammation, occasionally accompanied by sterile abscess formation, with or without mixed inflammatory infiltrates or lymphocytic vasculitis [2]. The evaluation of suspected PG should focus on excluding alternative etiologies. The diagnostic workup may include serologic testing for syphilis, antineutrophil cytoplasmic antibody (ANCA) testing to assess for vasculitis, a complete blood count, inflammatory markers, investigations for inflammatory bowel disease (IBD), and serum or urine protein electrophoresis when clinically indicated [2].

PG is a rapidly progressive, immune-mediated neutrophilic dermatosis that generally requires early initiation of systemic immunosuppressive therapy. Treatment is typically initiated with a fast-acting immunosuppressive agent, such as systemic corticosteroids (e.g., prednisone) or cyclosporine, while appropriate wound care remains an essential component of management due to the ulcerative nature of the disease [3]. Once disease progression has been controlled, clinicians should evaluate the need for additional steroid-sparing immunosuppressive therapy (e.g., mycophenolate mofetil) to maintain remission and minimize corticosteroid exposure [2].

The therapeutic approach to PG has evolved with the introduction of newer agents, particularly anti-tumor necrosis factor-α (TNF-α) therapies. In patients with concomitant IBD, sulfasalazine and other 5-aminosalicylate derivatives were historically used; however, an increasing proportion of patients now receive biologic therapies, which have demonstrated efficacy in both PG and the underlying IBD [7].

Clinical reassessment is recommended within 1-3 weeks after initiating therapy. An adequate treatment response is characterized by the absence of new ulcer development, cessation of progression of preexisting lesions, improvement of ulcer margins, and a decrease in perilesional erythema, reflecting effective control of the inflammatory process [2,3]. In our patient, treatment with oral prednisone at 1 mg/kg/day combined with topical pimecrolimus resulted in an approximately 50% reduction in the lesions within three weeks.

## Conclusions

PG should be considered in the differential diagnosis of chronic, recurrent, or rapidly progressive vulvar ulcerations in pediatric patients, especially when lesions fail to respond to antimicrobial therapy and infectious causes have been excluded. Given the rarity of vulvar involvement in children, diagnosis may be delayed, increasing the risk of unnecessary treatments and procedures. Histopathologic examination, although nonspecific, can provide important supportive evidence when interpreted in the appropriate clinical context. Early recognition and prompt initiation of immunosuppressive therapy are essential for disease control and may result in significant clinical improvement, as demonstrated in our patient, who achieved a 50% reduction in lesion burden within three weeks of treatment with oral prednisone and topical pimecrolimus.
